# Nutritional Intake and Gut Microbiome Composition Predict Parkinson’s Disease

**DOI:** 10.3389/fnagi.2022.881872

**Published:** 2022-05-11

**Authors:** Michal Lubomski, Xiangnan Xu, Andrew J. Holmes, Samuel Muller, Jean Y. H. Yang, Ryan L. Davis, Carolyn M. Sue

**Affiliations:** ^1^Department of Neurology, Royal North Shore Hospital, Northern Sydney Local Health District, St Leonards, NSW, Australia; ^2^Department of Neurogenetics, Faculty of Medicine and Health, Kolling Institute, University of Sydney and Northern Sydney Local Health District, St Leonards, NSW, Australia; ^3^School of Medicine, The University of Notre Dame Australia, Sydney, NSW, Australia; ^4^School of Mathematics and Statistics, Sydney Precision Bioinformatics, University of Sydney, Sydney, NSW, Australia; ^5^Charles Perkins Centre, University of Sydney, Sydney, NSW, Australia; ^6^School of Life and Environmental Sciences, University of Sydney, Sydney, NSW, Australia; ^7^Department of Mathematics and Statistics, Macquarie University, Sydney, NSW, Australia

**Keywords:** Parkinson’s disease, gut microbiota, gastrointestinal microbiome, dysbiosis, medication, biomarker, prediction model

## Abstract

**Background:**

Models to predict Parkinson’s disease (PD) incorporating alterations of gut microbiome (GM) composition have been reported with varying success.

**Objective:**

To assess the utility of GM compositional changes combined with macronutrient intake to develop a predictive model of PD.

**Methods:**

We performed a cross-sectional analysis of the GM and nutritional intake in 103 PD patients and 81 household controls (HCs). GM composition was determined by 16S amplicon sequencing of the V3-V4 region of bacterial ribosomal DNA isolated from stool. To determine multivariate disease-discriminant associations, we developed two models using Random Forest and support-vector machine (SVM) methodologies.

**Results:**

Using updated taxonomic reference, we identified significant compositional differences in the GM profiles of PD patients in association with a variety of clinical PD characteristics. Six genera were overrepresented and eight underrepresented in PD patients relative to HCs, with the largest difference being overrepresentation of *Lactobacillaceae* at family taxonomic level. Correlation analyses highlighted multiple associations between clinical characteristics and select taxa, whilst constipation severity, physical activity and pharmacological therapies associated with changes in beta diversity. The random forest model of PD, incorporating taxonomic data at the genus level and carbohydrate contribution to total energy demonstrated the best predictive capacity [Area under the ROC Curve (AUC) of 0.74].

**Conclusion:**

The notable differences in GM diversity and composition when combined with clinical measures and nutritional data enabled the development of a predictive model to identify PD. These findings support the combination of GM and nutritional data as a potentially useful biomarker of PD to improve diagnosis and guide clinical management.

## Introduction

Parkinson’s disease (PD) is a common progressive multisystem neurogenerative disorder (Rietdijk et al., 2017) that is associated with significant morbidity and healthcare burden, resulting in the deterioration of quality of life (QoL) (Lubomski et al., 2015, 2021b). Gastrointestinal dysfunction is a well-recognized prodromal non-motor symptom (NMS) in PD (Postuma et al., 2012; Lubomski et al., 2020b), with constipation and prolonged intestinal transit times evident many years prior to the development of classical motor symptoms (Savica et al., 2009; Pfeiffer, 2011). With GI dysfunction strongly linked to gut health and the microbiome, causal links have been established between the gut and the brain in PD, with the enteric nervous system (ENS) implicated in early pathogenesis prior to the central nervous system (Hawkes et al., 2007). Consequently, two subtypes of PD pathogenesis have been proposed, brain-first or body-first (Horsager et al., 2020).

There are numerous reports of variations in the gut microbial composition in PD patients, as well as associations to a variety of clinical disease outcome measures (Lubomski et al., 2020b; Romano et al., 2021). Interest in relationships between PD and the gut microbiome (GM) have increased since initial studies were published in 2015 (Hasegawa et al., 2015; Keshavarzian et al., 2015; Scheperjans et al., 2015). Meta-analyses (Nishiwaki et al., 2020; Romano et al., 2021; Shen et al., 2021) of these studies also suggest an enrichment and depletion of various taxa in association with PD. One mechanism thought to contribute to body-first PD pathogenesis is underrepresentation of short-chain fatty-acid (SCFA) producing bacteria considered important in maintaining gut function, integrity and health. Evidence suggests that gut dysbiosis in PD may drive gut and systemic inflammation, leading to impairment of host immune functions that underlie the prevalent gastrointestinal symptoms observed in patients with PD (Bullich et al., 2019; Romano et al., 2021).

More directly, there is evidence of microbe impacts in the gut that are mechanistically relevant to synucleinopathy (Sampson et al., 2016), potentially contributing to α-synuclein (α-syn) aggregation in the ENS that results in the accelerated caudo-rostral neurodegenerative spread observed in PD (Bullich et al., 2019; Lubomski et al., 2020). The GM has also been implicated in the variability of therapeutic outcomes, most notably the influence of *Enterococcus faecalis* on the metabolism of Levodopa (Maini Rekdal et al., 2019; van Kessel et al., 2019), which could serve as a modifiable target for improving Levodopa efficacy (Lubomski et al., 2019).

The aim of this study was to investigate associations between the GM and clinical parameters to identify relationships that could indicate the presence of PD. Associations between disease characteristics, therapeutic regimes, diet and the GM were explored and used to develop predictive models of PD that may eventually guide diagnosis and clinical management at earlier stages of PD.

## Materials and Methods

### Study Settings and Subjects

Consecutive PD patients presenting to the movement disorder and neurology clinics at Royal North Shore Hospital, Sydney, Australia, were recruited to this study as reported previously (Lubomski et al., 2020b,2021c). Patient inclusion criteria were (1) >18 years of age, (2) a clinical diagnosis of idiopathic PD according to the UK Parkinson’s Disease Society Brain Bank Diagnostic Criteria (Hughes et al., 1992), and (3) being managed by a specialist neurologist. Household controls (HCs) were opportunistically recruited when presenting to clinic with a PD patient. HC inclusion criteria were (1) >18 years of age, (2) self-reporting and displaying no obvious clinical signs of PD and (3) a spouse, sibling or child with similar dietary habits to their respective PD subject. Exclusion criteria included secondary Parkinsonism, tube feeding, medical or surgical disorders preventing completion of questionnaires and significant cognitive impairment demonstrated by incapacity to provide consent. All participants did not receive antibiotics or probiotic supplements for at least 1-month prior to sample collection.

Ethical approval was granted by the Northern Sydney Local Health District Human Research Ethics Committee (HREC/18/HAWKE/109) and the North Shore Private Hospital Ethics Committee (NSPHEC 2018-LNR-009). Written informed consent was obtained from all subjects at the time of recruitment.

### Clinical Data and Sample Collection

PD and HC participants attending clinics between June 2018 and June 2019 were recruited to complete questionnaires, as well as providing a stool (see below) and blood sample (Supplementary Figure 1). Non-fasting blood samples were assessed with standard pathology assays for liver function, non-fasting lipid profiles, Erythrocyte Sedimentation Rate and C-Reactive Protein, performed by NSW Pathology, Royal North Shore Hospital.

Information regarding socio-demographic factors, lifestyle, clinical management and comorbidities was collected from validated surveys, as previously reported (Lubomski et al., 2020a,b, 2021a,b,2021c; Palavra et al., 2021). A comprehensive Food Frequency Questionnaire (Barclay et al., 2008), was completed by all participants. Dietary questionnaires allowed for extrapolation of macronutrient intake, including energy, protein, fat, carbohydrate, sugar, fiber, moisture, and vegetarian status (Palavra et al., 2021). Patients completed validated clinical questionnaires assessing upper gastrointestinal symptoms [Leeds Dyspepsia Questionnaire (LDQ) (Moayyedi et al., 1998)], constipation severity and gut motility [Rome-IV criteria (Sood and Ford, 2016) and the Cleveland Constipation Scale (CCS) (Agachan et al., 1996)], QoL [PDQ-39 (Jenkinson et al., 1997) and the Short Form Health Survey (SF-36) (Ware and Sherbourne, 1992)], physical activity [International Physical Activity Questionnaire (IPAQ) (Hagstromer et al., 2006)], mood [Beck Depression Inventory (BDI) (Beck et al., 1961)], cognitive function [Montreal Cognitive Assessment (MoCA) (Nasreddine et al., 2005)], chronic pain severity (Visual Analogue Scale; McCormack et al., 1988) and non-motor symptoms [Non-Motor Symptoms Scale (NMSS) (Chaudhuri et al., 2007)]. Clinical motor assessments were performed by one neurologist (ML) during a patient’s “on” state, as an objective measure of the prevailing motor function, in accordance with the Movement Disorder Society—Unified Parkinson’s Disease Rating Scale—Part III (MDS-UPDRS III) criteria (Goetz et al., 2008). Medications were compared following standard methods for calculating daily levodopa equivalent dose (LED) (Tomlinson et al., 2010).

### Fecal DNA Extraction and 16S Ribosomal RNA Amplicon Sequencing

Stool samples were collected from 103 PD patients (including 27 device-assisted therapy (DAT) patients) and 81 HCs. Stool samples were collected into sterile pots, snap frozen and stored at −80°C within 24 h of collection. Upon receipt, stool samples were assessed against the Bristol Stool Scale (BSS) (Lewis and Heaton, 1997). Total fecal DNA isolation was carried out within 2 months of collection, using an optimized protocol for the MP Biomedicals FastDNA™ SPIN Kit for Feces (MP Biomedicals, Santa Ana, CA, United States), as reported previously (Lubomski et al., 2021c). DNA integrity was confirmed by polymerase chain reaction using universal primers to the V3–V4 regions (341f and 805r) and the whole rRNA gene (27f and 1492r) of bacterial 16S ribosomal DNA (Weisburg et al., 1991; Klindworth et al., 2013). Amplicons were separated by agarose gel electrophoresis to confirm the presence of an amplicon at the correct size.

16S rRNA V3-V4 amplicon sequencing was performed by the Ramaciotti Center for Genomics (University of New South Wales, Sydney, Australia). Sequencing libraries were generated using standard V3-V4 primers (341f and 805r; Weisburg et al., 1991) and a two-stage amplicon and indexing PCR with KAPA HiFi polymerase to generate 300 bp paired-end reads. Libraries were purified after each PCR using Ampure XP beads and normalized using the Applied Biosciences SequalPrep™ Plate Normalization kit (Thermo Fisher). Sequencing was performed on an Illumina MiSeq platform using MiSeq v3 chemistry with PhiX control v3. Internal sequencing controls included replicate patient stool DNA samples and the ZymoBIOMICS Microbial Community DNA Standard (Zymo Research, Irvine, CA, United States) for validation of sequencing and batch normalization.

### Computational and Statistical Analyses

Data was assessed by Levene’s test to determine homogeneity of variances. Clinical data comparisons between groups were performed using Student’s *t*-tests and χ^2^-tests (SPSS v.26 SPSS Inc., Chicago, IL, United States) for quantitative and categorical variables, respectively. Pairwise Spearman correlations assessed non-parametric associations between microbiota and clinical covariates. For all tests, a *p* < 0.05 was considered statistically significant. All microbiome statistical comparisons and data visualizations were performed with R (v.3.5.1) and figures were generated with ggplot2 (v.3.1.0).

#### Pre-processing

The R-package dada2 (v.1.14.1) was used to process sequence data into amplicon sequence variant (ASV) tables. The forward and reverse error profiles were trimmed to maintain high read quality (Supplementary Figure 2). The sequences were trimmed from 37 to 270 bp and 10 to 222 bp in forward and reverse reads, respectively. Subsequently, the sequence data was deduplicated to remove redundancy and combine all identical sequence reads into a “unique sequence.” Sequences were then denoised by removing substitution and indel errors. The resulting sequence was further merged by removing paired sequences without perfect overlap. Finally, the chimeras were removed by comparing each inferred sequence to others. ASVs were assigned to taxonomic groups according to Silva (v.138) reference database. After processing, a total of 9,479 ASVs were identified. For the ASV table, we selected all ASVs that were above the detection threshold for at least 10% (18 patients) of all participants individuals. The detection threshold was defined here as any presence of reads.

#### Microbiological Community Analysis

Alpha diversity metrics, including the Shannon index and taxon richness, were calculated for each sample, with a Wilcoxon test performed for differences between PD and HC groups. Beta diversity was used to assess turnover between samples with three commonly used metrics, i.e., Bray-Curtis (BC) dissimilarity, unweighted and weighted unifrac distance. All diversity indices were calculated using functions in the R-package vegan. A Principal Component Analysis (PCoA) was used for both dimension reduction and visualizing the relationships among samples. To assess the significance of beta diversity between cohorts, we used a permutational multivariate analysis of variance (PERMANOVA) model (adonis function implemented in the vegan package; (v.2.5-7) with the parameter “by” to margins and “perm” to 9,999 for all comparisons). To compare the compositional difference between the PD and HC groups, an ANOVA-like differential expression (ALDE) model (implemented in R package ALDEXx2; v1.16) was used at four taxonomic levels (phylum, order, family, and genus), setting the parameter “mc.samples” to 128 and “denom” to All.

The association of microbiota and clinical covariates was determined by calculating their pairwise Spearman correlations. Subsequent partial correlations were calculated to determine clinical and GM associations, whilst controlling for age, gender and Body Mass Index (BMI). For all microbiome analytics, the comparisons were performed with data assigned at four taxonomic levels (phylum, order, family, and genus).

#### Data Visualization and Resource

An interactive Shiny app called “PDBug” was developed to enable further detailed exploration of clinical and GM differences, as well as associations and relative abundances within the PD and HC cohorts. PDBug is publicly available from http://shiny.maths.usyd.edu.au/PDBug/.

#### Prediction Analysis

We used random forest (RF) analysis from the R-package randomForest (v1.6-14) to generate predictive models using microbiota and clinical covariates at different taxonomic levels to identify PD.

Prediction performance for PD was assessed using different models at different taxonomic levels. Model performance at each taxonomic level was determined by applying leave-one-out cross validation to calculate the area under the receiver operating characteristics curve (LOOCV-AUC). Subsequently, a two-stage model was constructed from both macronutrient intake and microbiota profile data. The first stage partitioned samples into two sub-cohorts based on macronutrient intake, with a cut-off value defined at the splitting point, corresponding to the decision tree with macronutrient intake as the only node. The split was based on the maximum information gain from the entire cohort versus splitting to two sub-cohorts. The second stage surveyed different RF model prediction of PD for each of the sub-cohorts. A number of two-stage models were generated using different macronutrients as partitioning nodes and calculating the corresponding LOOCV-AUC for each.

## Results

### Demographic, Clinical, and Nutrition Characteristics

103 PD patients and 81 HC’s were enrolled into the study (Supplementary Figure 1), as previously described (Lubomski et al., 2020b). Briefly, 56.3% of the PD participants were male with a mean age of 67.1 years (range 35–88, standard deviation [SD 12.2]), whilst two thirds of the HCs were female, with a mean age of 62.4 years (range 18–90, [SD 15.6], *p* = 0.001). All relevant demographic, clinical parameter and nutritional intake information has been reported previously for this cohort (Lubomski et al., 2020a,b, 2021a,b; Palavra et al., 2021) and is reproduced in Tables 1, 2 and Supplementary Data, where relevant to this study.

**TABLE 1:** Cohort demographic and clinical characteristics.

	Parkinson’s disease	Household control	Test statistic (df)	*p*-value
Number of patients (*n* =)*	103	81		
Age, (years) [SD, range]*	67.1 [12.2, 33–88]	62.4 [15.6, 18–90]	*t* = 2.3 (182)∧	0.023
Gender, (%)*			χ^2^ = 10.7 (1)^8^	0.001
Male	56.3	32.1		
Female	43.7	67.9		
Marital status, (%)*			χ^2^ = 4.2 (3)^8^	0.244
Married/*de facto*	76.7	85.1		
Single	9.7	9.9		
Widowed	5.8	1.2		
Other	7.7	3.7		
Ethnicity, (%)*			χ^2^ = 2.3 (3)^8^	0.506
Caucasian	78.6	79.0		
Asian	3.9	6.2		
Middle Eastern	6.8	2.5		
Other	10.7	12.3		
Body mass index, [SD] *	25.7 [5.2]	26.2 [4.6]	*t* = -0.7 (182)∧	0.485
Last antibiotic use (months), [SD, range] *	21.9 [33.8, 1–280]	25.8 [37.8, 1–288]	t = -0.7 (182)∧	0.475
Smoking history, (%)*				
Current smoker	1.9	3.7	χ^2^ = 0.6 (1)^8^	0.457
Prior smoker	36.9	33.7	χ^2^ = 0.2 (1)^8^	0.659
Pack year history, [SD]	13.3 [13.8]	14.4 [14.6]	*t* = -0.3 (63)∧	0.758
Alcohol consumption, (%)*	70.0	87.7	χ^2^ = 8.7 (1)^8^	0.003
< Weekly	23.5	27.2	χ^2^ = 0.3 (1)^8^	0.574
Several times weekly	31.1	33.3	χ^2^ = 0.8 (1)^8^	0.778
Daily	16.7	28.4	χ^2^ = 3.6 (1)^8^	0.057
Caffeine consumption (Coffee/Tea), (%)*	85.4	91.4	χ^2^ = 1.5 (1)^8^	0.219
Number of daily cups, [SD]	2.3 [1.7]	3.1 [1.8]	*t* = 3.0 (182)∧	0.003
History of diabetes, (%)*	4.9	6.2	χ^2^ = 0.2 (1)^8^	0.695
Gastrointestinal symptoms*				
Cleveland constipation score, [SD]	7.2 [4.7]	3.1 [2.9]	*t* = 6.9 (182)∧	<0.001
Constipation score as per Rome IV criteria, [SD]	4.4 [3.5]	1.1 [1.4]	*t* = 7.9 (182)∧	<0.001
Functional constipation as per Rome IV criteria, (%)	78.6	28.4	χ^2^ = 46.6 (1)^8^	<0.001
Bristol stool score, [SD]	2.8 [1.5]	3.9 [1.3]	*t* = 4.0 (182)∧	<0.001
Leeds dyspepsia questionnaire (LDQ) score, [SD]*	8.3 [7.7]	4.6 [6.1]	*t* = 3.5 (182)∧	0.001
Most troublesome symptom, (%)			χ^2^ = 15.2 (7)∧	0.034
Indigestion	18.4	8.6		
Heartburn	7.8	9.9		
Regurgitation	6.8	7.4		
Belching	7.8	6.2		
Nausea	15.6	7.4		
Vomiting	1	0		
Excess fullness/bloating	20.4	14.8		
None	22.3	45.7		
Chronic pain over last 3 months, (%)*	72.8	39.5	χ^2^ = 20.7 (1)^8^	<0.001
Pain score (visual analog scale), [SD]	4.9 [2.5]	3.9 [1.7]	*t* = 2.0 (105)∧	0.046
International physical activity questionnaire (IPAQ) score (MET-minutes/week), [SD]*	1823.6 [1693.6]	2942.4 [2620.9]	*t* = -3.5 (182)∧	0.001
IPAQ categorical score, (%)			χ^2^ = 7.1 (2)^8^	0.029
Low	35.2	19.8		
Moderate	37.9	39.6		
High	26.2	40.7		
Sitting hours/day, [SD]	6.5 [3.5]	4.8 [2.3]	*t* = 3.7 (182)∧	<0.001
Able to walk 1 km, (%)	73.8	97.5	χ^2^ = 19.3 (1)^8^	<0.001
Able to climb 1 flight of stairs, (%)	86.4	100	χ^2^ = 11.9 (1)^8^	0.001
Biochemical characteristics, [SD]*				
Erythrocyte sedimentation rate (mm/h)	9.5 [13.4]	9.5 [10.4]	*t* = -0.1 (181)∧	0.991
C-reactive protein (mg/L)	3.9 [10.8]	2.2 [2.5]	*t* = 1.4 (182)∧	0.177
Total cholesterol (mmol/L)	4.8 [0.9]	5.2 [1.1]	*t* = -2.5 (182)∧	0.014
Low density lipoprotein (mmol/L)	2.7 [0.7]	2.9 [0.9]	*t* = -1.5 (178)∧	0.132
High density lipoprotein (mmol/L)	1.4 [0.4]	1.6 [0.4]	*t* = -2.2 (181)∧	0.033
Trigl ycerides (mmol/L)	1.3 [1.0]	1.5 [0.9]	*t* = -1.2 (182)∧	0.239
Random glucose (mmol/L)	5.8 [0.6]	5.9 [0.9]	*t* = -0.8 (182)∧	0.438
HbA1c%	5.3 [0.4]	6.0 [5.2]	*t* = -1.2 (182)∧	0.217
Albumin (g/L)	38.7 [3.5]	39.8 [3.1]	*t* = -2.3 (182)∧	0.023
Dietary intake				
Vegetarian diet, (%)	2.9%	2.5%	χ^2^ = 0.1 (1)∧	0.865
Energy (kJ/day), [SD]	11130.9 [5782.6]	10188.2 [4799.9]	*t* = 1.2 (181)∧	0.241
Protein (g/day), [SD]	118.4 [79.3]	116.7 [74.5]	*t* = 0.1 (181)∧	0.883
Fat (g/day), [SD]	101.7 [49.7]	95.7 [43.6]	*t* = 0.9 (181)∧	0.392
Carbohydrate (g/day), [SD]	278.8 [161.8]	232.2 [124.8]	*t* = 2.1 (181)∧	0.034
Total sugars (g/day), [SD]	153.3 [86.3]	118.7 [60.6]	*t* = 3.0 (181)∧	0.003
Fiber (g/day), [SD]	41.1 [31.2]	38.1 [22.7]	*t* = 0.7 (181)∧	0.475
Moisture (mL/day), [SD]	2877.9 [1236.2]	3044.3 [1050.6]	*t* = -0.1 (181)∧	0.337
Depression characteristics				
Beck’s depression inventory total score, [SD]	11.9 [8.8]	5.2 [5.5]	*t* = 5.9 (182)∧	<0.001
Beck’s depression inventory categories, (%)			χ^2^ = 25.2 (3)^8^	<0.001
Minimal depression (0–13)	64.1%	95.1%		
Mild depression (14–19)	19.4%	2.5%		
Moderate depression (20–28)	10.7%	1.2%		
Severe depression (39–63)	5.8%	1.2%		
Clinically depressed, (> 13 for Parkinson’s disease and > 9 for control groups), (%)	38.9%	20.1%	χ^2^ = 6.8(1)^8^	0.009
Montreal cognitive assessment (MoCA), [SD]				
MoCA total score, (/30)	24.4 [4.8]	27.6 [2.5]	*t* = -5.4 (182)∧	<0.001
Mild cognitive impairment (< 26/30), (%)	48.6	18.5	χ^2^ = 17.9 (1)^8^	<0.001
Parkinson’s disease dementia (< 21/30), (%)	16.5	-		
36—Item short form health survey (quality of life assessment), [SD]				
Health change over last year	38.8 [21.7]	50.6 [16.3]	*t* = -4.0 (182)∧	<0.001
Physical component summary	51.6 [22.7]	79.9 [17.7]	*t* = -9.3 (182)∧	<0.001
Mental component summary	60.9 [22.2]	80.8 [17.4]	*t* = -6.6 (182)∧	<0.001

∧(Independent Sample t-test), ^8^(Pearson’s chi-squared test), df (degrees of freedom), SD, (Standard Deviation). *This data is partially reproduced (Lubomski et al., 2020b).

**TABLE 2:** Parkinson’s disease clinical characteristics.

Age at diagnosis, (years) [SD, range]*	58.8 [13.6, 24–88]
Parkinson’s disease duration, (years) [SD, range]*	9.2 [6.5, 1–30]
Parkinson’s disease phenotype, (%)*	
Tremor Dominant	30.1
Postural Instability and gait Impairment	20.4
Akinetic rigid	38.9
Young onset (<40years)	10.7
Late onset (>60years)	49.5
Genetic diagnosis, (%)*	1.9
Disease complications, (%)*	
Motor fluctuations	58.3
Dyskinesia	58.3
Wearing off	81.6
Impulse control disorder	19.4
Non-motor symptoms, (%)*	
Hyposmia	73.8
REM sleep behavior disorder	48.5
Constipation	60.2
Levodopa equivalent daily dose (mg), [SD, range]*	834.8 [527.3, 0–2,186]
MDS unified Parkinson’s disease rating scale-III (“on” state), [SD, range]*	32.9 [17.7, 5–91]
Quality of life	
PDQ-39 summary index, [SD]	29.2 [17.3]
MDS Non-motor symptoms score (NMSS)—total score, [SD]*	62.7 [42.9]
Parkinson’s disease therapy, (%)*	
Treatment naïve	(*n* = 5) 4.9
Oral levodopa	(*n* = 92) 89.3
Dopamine agonist	(*n* = 36) 35.0
Monoamine oxidase B inhibitor	(*n* = 19) 18.4
Anticholinergic	(*n* = 13) 12.6
Catechol-O-methyl transferase inhibitor	(*n* = 24) 23.3
Amantadine	(*n* = 13) 12.6
Levodopa-carbidopa intestinal gel (LCIG)	(*n* = 9) 8.7
Deep brain stimulation	(*n* = 11) 10.7
Apomorphine (subcutaneous infusion)	(*n* = 7) 6.8

SD, (Standard Deviation). *This data is partially reproduced (Lubomski et al., 2020b).

### Microbiome Data for Analysis

The total number of sequencing reads was 11,927,248, with a mean of 64,822 reads per sample that were assigned to 9,479 ASVs. After filtering low abundance-ubiquity (<10% of the sample or appeared in less than 18 samples), the final dataset was represented by 627 ASVs. These ASVs were assigned to 9 phyla, 31 orders, 48 families and 138 genera. The most abundant taxa were similar for the PD and HC groups, with the order *Clostridiales* being the most represented in the two groups. This is one of the first PD GM studies utilizing the new taxonomic reference, which provides a more detailed sub-classification of taxonomic profiles giving increased resolution of bacterial community composition. Thus, the bacterial names ascribed here may differ from previous PD GM studies, but where relevant consistencies have been highlighted.

### Differences in the Gut Microbiota Between the Parkinson’s Disease and Household Control Cohorts

#### Diversity

**FIGURE 1:**
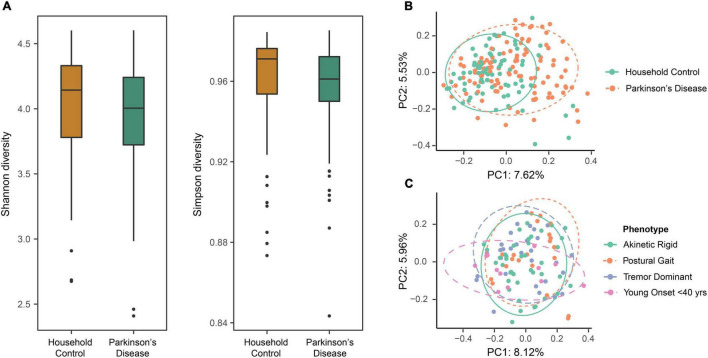
The evaluation of diversity measures between the household control (HC) and Parkinson’s disease (PD) groups identified differences in beta diversity measures but not alpha diversity. (A) Box plots representing alpha diversity showed no significant differences in Shannon (species abundance and evenness within a community) or Simpson (species richness and evenness within a community) diversity between the HC and PD cohorts (ANOVA, *p* = 0.057 and 0.159, respectively). (B) Beta diversity using principal coordinate analysis (PCoA) with Bray-Curtis dissimilarity at amplicon sequence variant (ASV) level. Comparison of the first two principal components revealed varied beta diversity (extent of species diversity difference between two environments) between the groups (PERMANOVA, *p* < 0.0001), suggestive of a disease-related effect on GM composition that might define a PD-related GM profile. Colored ellipses (solid green = HC and dotted orange = PD) represent a 90% confidence region and the proportion of total variance represented by a given principal component is labeled on the respective axis. (C) Evaluating the effects of PD phenotypes in terms of gut microbial beta diversity showed no overall statistical significance between the four groups (PERMANOVA, *p* = 0.112). Although, the greatest diversity difference was seen for the younger onset < 40 years subgroup, as compared to the tremor dominant, akinetic rigid and postural instability subgroups.

Alpha diversity (partitioning of biological space in each community) was assessed by Shannon and Simpson diversity indices to compare the two cohorts. No significant difference in the alpha diversity between the PD and HC groups was observed at the ASV taxonomic level (ANOVA, *p* = 0.057 and 0.159 for Shannon and Simpson diversity, respectively) (Figure 1A).

Beta diversity was analyzed separately using PCoA with BC dissimilarity, unweighted unifrac and weighted unifrac. Exploring the relationships between the PD and HC groups (*n* = 184) at the ASV level, BC ordination showed a significant difference between the two groups (PERMANOVA, *p* < 0.0001). Principle Components (PC) 1 and 2 showed a clustering of beta diversity for the HC group compared to the more widely distributed PD cohort (Figure 1B). Further, beta diversity differences were evaluated between the various PD phenotypes, but overall no statistically significant differences between the four groups were apparent (PERMANOVA, *p* = 0.112). Although, a trend was observed that suggested most of the diversity change was within the younger onset (<40 years of age) subgroup compared to the tremor dominant, akinetic rigid and postural instability subgroups (Figure 1C).

#### Relative Abundance

**FIGURE 2:**
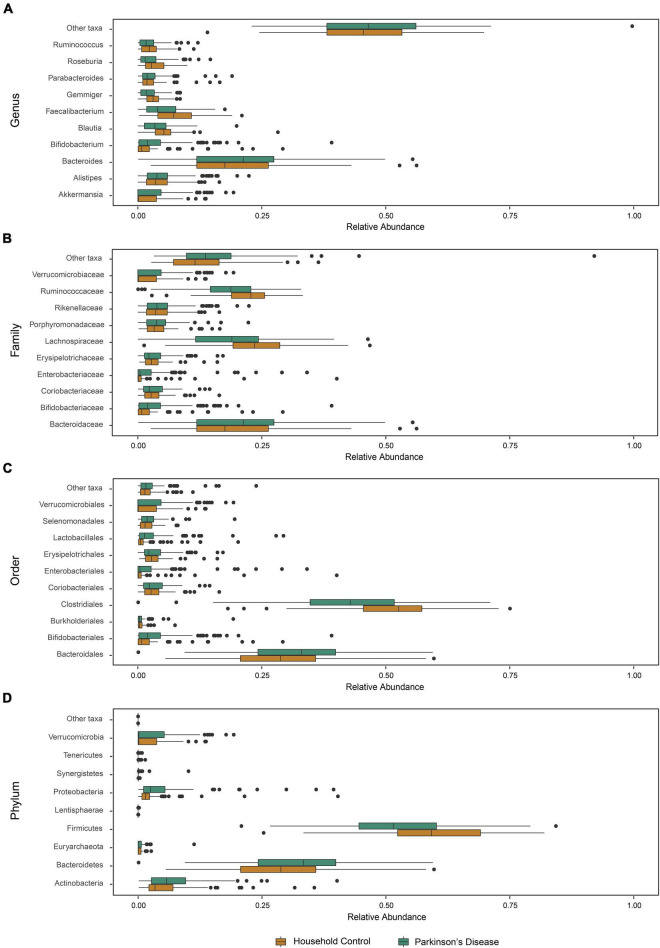
Microbiota abundance for household control (HC) and Parkinson’s disease (PD) groups. The relative abundance of phylogenetic gut microbiome taxa composition at the (A) genus, (B) family, (C) order, and (D) phylum level for individual participants (*n* = 81 HCs and *n* = 103 PD) showed a statistically significant compositional difference between PD and HC groups at each studied taxonomic level (PERMANOVA, *p* < 0.01 genus, *p* < 0.01 family, *p* < 0.01 order, *p* = 0.02 phylum).

Given the broader diversity considerations of the cohorts, we then examined characteristics of microbiome community structure in the PD and HC cohorts. Comparison of the mean taxon compositions between PD and HC cohorts across different taxonomic ranks are presented in Figure 2. At each taxonomic level analyzed, a statistical difference in the mean relative abundance was noted between the PD and HC groups (PERMANOVA, *p* < 0.01 genus, *p* < 0.01 family, *p* < 0.01 order, *p* = 0.02 phylum taxonomic levels). The relative abundance differences at the family taxonomic level for each individual PD and HC participant are presented in Supplementary Figure 3. These results highlight the innate variability of GM composition across the cohorts, within which we identified cohort-specific differences in relative abundance.

#### Exploration of Composition Differences for Indicator Taxa

**FIGURE 3:**
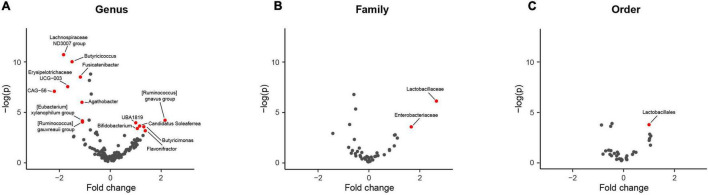
Comparison of taxa abundance between household (HC) and Parkinson’s disease (PD) patients at different phylogenetic levels reveals specific differences. Volcano plots representing abundance differences (fold change) of different taxa between HC and PD patients showed statistically significant [-log (*p*) > 3; fold change > ± 1.2] compositional differences at the genus, family and order levels (represented by red dots), indicative of a PD-related GM composition. With regards to PD patients, there was statistically significant overrepresentation of *Bifidobacterium*, *Candidatus Soleaferre, Butyricimonas, Flavonifractor, [Ruminococcus] gnavus group*, and *Faecalibacterium* sp. *UBA1819* and underrepresentation of *Butyricicoccus, Fusicatenibacter, Lachnospiraceae ND3007 group*, *Erysipelotrichaceae UCG-003*, *Agathobacter*, *[Eubacterium] xylanophilum group*, *[Ruminococcus] gauvreauii group*, and *Firmacutes bacterium CAG:56* at the genus level (A), overrepresentation of *Lactobacillaceae* and *Enterobacteriaceae* at the family level (B) and overrepresentation of *Lactobacillales* at the order level (C). The largest fold change was observed for increased *Lactobacillaceae* taxa abundance (2.7 fold increase).

Comparing differences in relative abundance for specific taxa between PD and HC groups with the ALDEx model, statistically significant compositional differences at the order, family and genus levels were apparent (Figure 3). Further supporting divergent GM profiles in PD patients. The largest difference of 2.7-fold was observed for increased *Lactobacillaceae* abundance at the family level, consistent with overrepresentation of *Lactobacillales* at the order level (Table 3). Six genera were found to be overrepresented and eight underrepresented in PD patients relative to HCs, with variable numbers of ASVs contributing to the abundance differences in the identified genera (Table 3).

**TABLE 3:** Gastrointestinal microbiota compositional differences between Parkinson’s Disease patients and Household Controls.

Overrepresented in PD cases			Underrepresented in PD cases
Order	Family	Genus	Genus
*Lactobacillales*	*Enterobacteriaceae* *Lactobacillaceae*	*Bifidobacterium* [9] *Butyricimonas* [5] *Candidatus Soleaferre* [2] *Faecalibacterium* sp. *UBA1819* [1] *Flavonifractor* [2] *[Ruminococcus] gnavus group* [1]	*Agathobacter* [4] *Butyricicoccus* [6] *Erysipelotrichaceae UCG-003* [2] *[Eubacterium] xylanophilum group* [3] *Fusicatenibacter* [1] *Lachnospiraceae ND3007 group* [1] *[Ruminococcus] gauvreauii group* [2] *Firmacutes bacterium CAG:56* [1]

*Square brackets indicate the number of ASVs contributing to the compositional difference in each genera.*

### Associations of Gut Microbiota Characteristics Within the Parkinson’s Disease Cohort

#### Clinical Features of Parkinson’s Disease and Beta Diversity

The association of various clinical characteristics with beta diversity in the PD cohort was examined by PERMANOVA. Constipation severity (Rome-IV criteria, *p* = 0.001; CSS, *p* = 0.022; BSS, *p* = 0.027), cognitive impairment (MoCA total score, *p* = 0.041; Mild Cognitive Impairment, *p* = 0.017), physical activity (IPAQ score, *p* = 0.017), pain (chronic pain, *p* < 0.001; pain severity, *p* < 0.001) and pharmacological therapies (Levodopa, *p* = 0.030; COMT inhibitor, *p* = 0.009) all showed a statistically significant association with changes in beta diversity. These clinical variables highlight a broad spectrum of influences on the GM species richness in PD patients. Five PD, but did not show a statistically different beta diversity from the rest of the PD cohort (Supplementary Figure 4).

#### Correlation Analyses

Partial correlation analysis, adjusting for patient age, sex and BMI, was performed to further evaluate significant associations between clinical variables and GM composition as indicated by Spearman correlations (Supplementary Table 1). Supplementary Table 1 describes many of the clinically relevant and statistically significant GM and PD motor and non-motor correlations. The majority of statistically significant correlations were weak (*r*_*s*_ = 0.2–0.4), with only 10 comparisons having a moderate correlation (*r*_*s*_ = 0.4–0.6) and three borderline moderate correlations (*r*_*s*_ = 0.398). Relevant correlations of clinical interest are discussed below.

Due to the sheer number of possible correlative combinations, we developed a comprehensive and interactive interface^1^ to allow clinicians and researchers to interrogate the entire data set of microbiome and clinical variables, with capacity to adjust the analysis for potential confounding factors.

#### Device-Assisted and Standard Parkinson’s Disease Therapies

When comparing relative bacterial abundances in the device-assisted PD therapy sub-cohorts [levodopa-carbidopa intestinal gel (LCIG) *n* = 9, DBS *n* = 11, Apomorphine *n* = 7], a number of weak correlations reaching statistical significance were apparent (Supplementary Table 1). Two moderate positive correlations were identified between LCIG therapy and the bacteria *Enterococcus* (*r*_*s*_ = 0.531, *p* = 0.01) and *ASV_155/Klebsiella* spp. (*r*_*s*_ = 0.411, *p* < 0.001), warranting further investigation of the validity and specificity of these associations with this particular therapy, given that it has a sub-physiological pH and is delivered directly into the upper GI tract. Separate and distinct correlative associations were identified for PD patients receiving standard therapies (Levodopa *n* = 92, Anticholinergics *n* = 13, COMT inhibitors *n* = 24, Amantadine *n* = 13, Dopamine agonists *n* = 36, MAO-B inhibitors *n* = 19) (Supplementary Table 1), with borderline moderate correlations between *ASV_166/Bifidobacterium* spp. and Anticholinergics (*r*_*s*_ = 0.398, *p* < 0.001), as well as *ASV_82/Lactobacillus* spp. and daily Levodopa Dose Equivalence (*r*_*s*_ = 0.398, *p* < 0.001).

#### Specific Associations Between Clinical Features and Gut Microbiota

While a considerable number of statistically significant but weak correlations were apparent for a wide range of clinical features, only a few had a moderate correlation coefficient (Supplementary Table 1). The physical component score of the SF-36 assessment gives an indication of overall physical health and was found to positively correlate with *Fusicatenibacter* (*r*_*s*_ = 0.444, *p* < 0.001), *Butyricicoccus* (*r*_*s*_ = 0.438, *p* < 0.001) and *ASV_32/Blautia* spp. (*r*_*s*_ = 0.401, *p* < 0.001). Consistent with the known interplay between gut function and dysbiosis, the BSS had a positive correlation with *Butyricicoccus* (*r*_*s*_ = 0.428, *p* < 0.001) and a borderline moderate association with *ASV_350/Clostridium_XIVa* spp. (*r*_*s*_ = 0.398, *p* < 0.001). Additionally, the ROME-IV Score showed an association with *ASV_151/Bacteroides* spp. (*r*_*s*_ = 0.411, *p* < 0.001), and the Leeds Dyspepsia Score correlated with both *Desulfomicrobiacaea* (*r*_*s*_ = 0.438, *p* < 0.001) and *Desulfomicrobium* (*r*_*s*_ = 0.422, *p* < 0.001). Lastly, a moderate correlation in a small subgroup of PD patients with Asian ethnicity (*n* = 4) and *ASV_173/Parasutterella* spp. (*r*_*s*_ = 0.489, *p* < 0.001) was identified, although this inference is underpowered in the current cohort.

Of the numerous weak associations between the GM and clinical measures, a number of important considerations were evident but require validation in larger cohorts. These include but are not limited to *Lactobacillaceae* and *Lactobacillus* as potential indicators for increased PD severity and duration (*r*_*s*_ = 0.257, *p* < 0.010) and (*r*_*s*_ = 0.323, *p* < 0.001) respectively. Lastly, other varied taxa suggested associations with numerous NMS, namely depression, chronic pain, RBD, in addition to demographics, dietary markers, and other influences from PD therapies that are seldom reported in the PD GM literature (Supplementary Table 1).

### Predictive Modeling of Gut Bacteria and Macronutrient Intake to Identify Parkinson’s Disease

**FIGURE 4:**
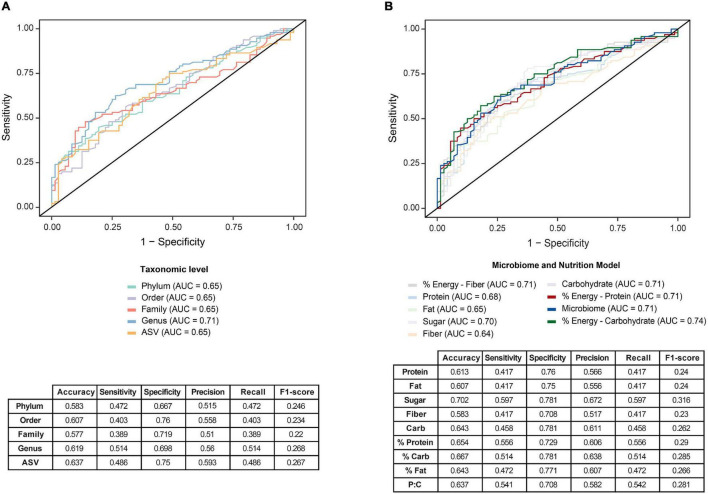
Predictive modeling to identify Parkinson’s disease (PD) was optimized by a two-stage model that incorporates nutritional and microbiome data. **(A)** Predictive Random Forest modeling was undertaken to identify the utility of the gut microbiome as a potential signature for PD. Comparisons at five taxonomic levels; phylum, order, family, genus and ASV, were used to predict PD, with greatest predictive capacity provided at the genus level on receiver operating characteristic curve (ROC), with an area under the curve (AUC) of 0.71. **(B)** An optimized two-stage predictive Random Forest model analysis was subsequently undertaken that considered dietary intake as an influence on the gut microbiome. Comparing the utility of the one-stage microbiome model (AUC = 0.71) with the two stage model, a slightly improved predictability was achieved. when incorporating dietary macronutrient data. Specifically, the incorporation of carbohydrate contribution to total energy in the model improved the prediction (AUC = 0.74), whereas incorporation of fiber, fat or protein macronutrient data alone into the model did not improve the predictive potential to identify PD. Accompanying sensitivity and specificity analyses are presented in the tables.

We examined the utility of the entire GM as a predictive signature of PD, developing two models using RF and support-vector machine (SVM) methodologies. The area under the curve (AUC) of receiver operating characteristic (ROC) curves were used to evaluate the predictive capacity of the model at different taxonomic levels. The RF model showed the best performance across all taxonomic levels and was used to identify the contribution of the GM to PD. Figure 4A illustrates the predictive capacity of the model at different taxonomic levels, with the highest AUC of 0.71 at the genus level.

As diet has a large influence on the GM, the RF model was then expanded to incorporate macronutrient data, with maximal information gain set to apportion the nutrient data for the best prediction of PD. The splitting nodes for each of the macronutrients included: protein, 127.77; fat, 85.3; added sugar, 38.1; fiber, 37.53; total available or digestible carbohydrate 163.07; percentage of energy intake as protein, 15%; percentage of energy intake as fat, 38% and percentage of energy intake as digestible carbohydrate, 38%. Accordingly, the incorporation of carbohydrate contribution to total energy, was shown to provide an improvement to the predictive ability of the model at the genus level to an AUC of 0.74 (Figure 4B). This two-stage model uniquely highlights the importance of incorporating clinical variables, particularly nutritional data, to define potential multivariate biomarkers of PD. These inferences require validation in a larger PD GM study and/or meta-analysis. The approach of using the global microbiome signature paired with important clinical measures to identify disease has been developed into a methodological approach pairing nutritional intake with gut enterotypes to define health state (Xu et al., 2021).

## Discussion

In this cross-sectional PD GM study, we enrolled a broad clinical spectrum of PD patients, representative of all stages of established PD and uniquely analyzed the influences of DATs, namely DBS and Apomorphine infusions, which have not been reported. When considering the entire GM in our analysis, we identified fourteen genera, two family and one order level variations in GM composition of PD patients when compared to HCs (Table 3 and Figure 3). Through our holistic consideration of the entire GM and extensive clinical parameters, we identified an array of GM associations with various therapeutic, motor and non-motor features. We developed predictive models to identify PD using the GM as a non-invasive fecal biomarker. These study findings provide further experimental evidence in support of GM involvement in body-first PD pathogenesis and disease progression. Iterative development of the foundational models from this study may eventually provide diagnostic indications at earlier stages of PD.

An increasing number of studies, mainly from Western Europe, China, Japan and the United States, have highlighted important patterns in the GM profiles of PD patients (Gorecki et al., 2019; Elfil et al., 2020; Lubomski et al., 2020), with more than 110 differentially abundant taxa described from the level of phylum to ASV or species (Boertien et al., 2019; Bullich et al., 2019; Lubomski et al., 2020; Wallen et al., 2020; Kenna et al., 2021; Romano et al., 2021). Although notable differences exist between the studies (study design, inclusion criteria, ages, gender proportions, disease durations, methodology, etc.), emerging consistencies for PD-specific GM changes suggest enrichment of the genera *Lactobacillus*, *Akkermansia*, and *Bifidobacterium* and depletion of bacteria belonging to the *Lachnospiraceae* family and the *Faecalibacterium* genus, as the most consistent PD GM alterations (Romano et al., 2021). One of the pitfalls for previous studies has been to consider only those differentially abundant taxa identified when creating disease discriminant models. We instead performed analyses by considering the entire GM and a diverse range of clinical measures to develop our disease-discriminant models.

The general species diversity across the cohort was not dissimilar, as indicated by a lack of statistical significance in alpha diversity. When comparing species richness between PD and HC groups, the statistical significance in beta diversity was supportive of a PD-specific GM profile. Furthermore, beta diversity differences were identified in association with constipation (Rome-IV criteria and CSS), cognitive profiles (MoCA Total Score), physical activity (IPAQ score), chronic pain, utilization of levodopa and COMT inhibitor medications, as well as an apparent trend for the young onset PD phenotype. This supports the multifactorial nature of the GM and disease interplay and indicates that a more global consideration of GM and clinical parameters is necessary. Of note, the longer mean disease duration of 9.2 years in this study, compared to many earlier PD GM studies, may explain why beta diversity changes were associated with so many clinical variables in our PD cohort, as divergence in beta diversity increases with disease progression (Barichella et al., 2018). Other studies have identified beta diversity differences associated with male sex, RBD, smoking and body-mass-index (Heinzel et al., 2020), although these were not apparent in the current study.

Consistent with beta diversity differences, a number of statistically significant relative abundance differences between the PD and HC groups were apparent, defining a PD-specific GM profile. At the family level, increased abundances of *Lactobacillaceae* (Scheperjans et al., 2015; Hill-Burns et al., 2017; Hopfner et al., 2017; Aho et al., 2019; Barichella et al., 2019; Pietrucci et al., 2019; Nishiwaki et al., 2020; Tan et al., 2021) and *Enterobacteriaceae* (Unger et al., 2016; Lin et al., 2018; Barichella et al., 2019; Pietrucci et al., 2019) identified here (Table 3), are consistent with reports from several earlier studies. At the genera level, overrepresentation of *Bifidobacterium* (Unger et al., 2016; Hill-Burns et al., 2017; Petrov et al., 2017; Aho et al., 2019; Barichella et al., 2019; Wallen et al., 2020) and *Butyricimonas* (Heintz-Buschart et al., 2018; Jin et al., 2019; Ren et al., 2020) and underrepresentation of *Lachnospiraceae ND3007 group* (Nishiwaki et al., 2020; Romano et al., 2021) identified here, have been repeatedly associated with PD in prior studies. The underrepresentation of *Butyricicoccus* and *Fusicatenibacter* in our cohort have been variably reported (Weis et al., 2019; Wallen et al., 2020), and in one study of Chinese PD patients, *Butyricicoccus* was reported to be overrepresented (Qian et al., 2018). This highlights the evident inconsistency across GM studies in PD and emphasizes the difficulties in differentiating between genuine inherent biological variability in small sample sizes and inconsistent study methodology. To our knowledge, the differentially represented genera *Candidatus Soleaferre, Flavonifractor*, *Faecalibacterium* sp. *UBA1819*, *Erysipelotrichaceae UCG-003*, *[Eubacterium] xylanophilum group*, *[Ruminococcus] gauvreauii group*, and *Firmacutes bacterium CAG:56* identified here, have not previously been described in association with a PD-specific GM profile and warrant consideration in subsequent studies. While we acknowledge the significant variation between studies, a recent consideration is the evolving taxonomic assignment nomenclature used to assign taxa from ASVs, which makes comparison with older studies more difficult but will improve the resolution of GM taxonomy and analysis in future studies.

A considerable body of evidence suggests underrepresentation of typically abundant SCFA-producing bacteria in PD can lead to SCFA imbalances that may have detrimental impacts on disease progression, including increased colonic inflammation, gut leakiness, risk of α-syn deposition in the gastrointestinal tract, and microglial activation in the brain (Bullich et al., 2019; Romano et al., 2021). In this study, we found overrepresentation of *Bifidobacterium* and *Butyricimonas* in our PD cohort. *Bifidobacterium* produces acetate and formate by metabolizing carbohydrates in plants and dairy (Wallen et al., 2020). They are a common component of probiotic supplements and have been shown to induce remission when supplemented in patients with Inflammatory Bowel Disease (Parada Venegas et al., 2019). On the other hand, *Butyricimonas* produces butyrate, which has been shown to reduce inflammation and maintain gut health (Parada Venegas et al., 2019). The increased relative abundance of these bacteria may be a protective attempt in response to systemic PD pathogenesis or the accompanying underrepresentation of other SCFA-producing bacteria. The underrepresented genera *Butyricicoccus*, *Fusicatenibacter* and *Lachnospiracaea ND3007 group* identified in this study all produce butyrate (Geirnaert et al., 2014), with *Lachnospiraceae ND3007 group* also producing acetate and alcohols (Boutard et al., 2014) that are believed to collectively exert anti-inflammatory effects, helping to maintain integrity of the gut membrane (Keshavarzian et al., 2020; Nishiwaki et al., 2020; Wallen et al., 2020). While the impact of variability in SCFA-producing bacteria in PD is still not entirely clear (Mulak, 2018; Dalile et al., 2019), there is strong suggestion of a role in pathogenesis and potential as therapy (Metzdorf and Tonges, 2021).

We identified many clinically important associations between the GM and clinical measures. With respect to DATs, after controlling for age, gender and BMI, two moderate correlations were identified between *Enterococcus* and *ASV_155/Klebsiella* spp and LCIG use. Associations with overrepresentation of *Enterococcus* signifies important implications for patients using large doses of levodopa and may result in positive selection of bacterium with tyrosine decarboxylase activity that can convert levodopa to dopamine in the gut (Lubomski et al., 2019; Maini Rekdal et al., 2019; van Kessel et al., 2019). This has implications for the treatment efficacy of levodopa administration in PD and could be exploited to improve dose efficiency. LCIG use was also associated with overrepresentation of *Enterobacteriaceae* and *Klebsiella* spp., which are genera of the family *Enterobacteriaceae* that is also independently associated with increased daily LED, Hoehn, and Yahr scores and motor fluctuations, consistent with motor disease severity, as previously identified (Li et al., 2017). The influence of DBS has been sparsely reported in regards to the GM (Lubomski et al., 2021c), but was associated here with overrepresentation of *Streptococcaceae* and *Streptococcus* spp. and underrepresentation of *Rikenellaceae*, potentially suggestive of peripheral effects of DBS on the GM that warrant further investigation. Uniquely, we identified positive associations between continuous subcutaneous Apomorphine infusion (a DAT that has not previously been studied in terms of the GM in PD) and the genera *Intestinibacter*, *Parasutterella*, and *Actinomyces*, again justifying further investigation.

In terms of standard therapies, oral Levodopa use was associated with the families *Sutterellaceae* and *Rikenellaceae* in our cohort, rather than *Bacillaceae*, as was reported in another study (Heintz-Buschart et al., 2018). Anticholinergic use was associated with *ASV_166/Bifidobacterium* spp., a beneficial bacterium known to digest dietary fiber and maintain healthy gut function (Castelli et al., 2021), although its association with this therapy has not been previously reported. Furthermore, COMT inhibitor use was associated with overrepresentation of *Bifidobacteriaceae*, *Enterococcaceae*, and *Lactobacillaceae* in our cohort. This is contrary to a previous report of a negative association with *Lachnospiraceae* abundance (Hill-Burns et al., 2017), although the relationships between the use of COMT inhibitors and gut microbiota have yielded many discordant findings in several other studies (Unger et al., 2016; Hill-Burns et al., 2017; Barichella et al., 2019). Furthermore, overrepresentation of *Lactobacillus* and *Lactobacillaceae* have often been seen as indicators of increased PD severity and duration, associating with PD duration, UPDRS-III total score, Hoehn and Yahr score, daily LED, adjuvant therapies for advanced disease (like COMT inhibitors and apomorphine), as well as decreased QoL, physical activity and increased depression severity (Lubomski et al., 2020b; Nishiwaki et al., 2020; Romano et al., 2021). Barichella et al. (2019) also identified a significant relationship between increased *Lactobacillaceae* and UPDRS-III total score. *Lactobacillaceae* overrepresentation in more advanced disease may be a relative finding in light of the underrepresentation of other genera, particularly from the family *Lachnospiraceae*, known for their beneficial SCFA production (Romano et al., 2021).

In terms of NMS in our PD cohort, better physical health, indicated by a higher physical component score from the SF-36 assessment, showed positive associations with *Fusicatenibacter*, *Butyricicoccus*, and *ASV_32/Blautia* spp. Whilst the clinical significance of these taxa associations are still unclear, important implications arising from favorable SCFA metabolism could be implicated (Aho et al., 2021), and warrants further study. Depression severity was positively associated with *Veillonella*, *Klebsiella*, and *Pseudoflavonifractor*, taxa that have not previously been described in association with mood changes in PD. Chronic pain was negatively associated with *Enterobacteriaceae*, whilst positively with *Bacteroidaceae* and *Synergistaceae* abundances, although these NMS have not been widely studied in prior PD GM studies. GI dysfunctions, practically constipation severity inferred from the BSS score and ROME-IV score, were most markedly associated with *Butyricicoccus* and *ASV_151/Bacteroides* spp., whilst other taxa including *ASV_350/Clostridium_XIVa* spp., *Faecalibacterium, Coprococcus*, and *Roseburia* spp. were also implicated. These bacteria have also previously been associated with chronic constipation in the general population (Zhao and Yu, 2016), although are different from some earlier PD studies reporting associations with *Bradyrhizobiaceae, Verrucomicrobiaceae* (Scheperjans et al., 2015), and *Bifidobacteria* (Baldini et al., 2020) in constipated PD patients. Distinctively, we also show moderate associations between upper GI dysfunction with overrepresentation of *Desulfomicrobiaceae* and *Desulfomicrobium*, associations not previously reported with regard to PD GM profiles.

Environmental influences of geography and dietary habits are known to be highly influential on GM composition. We made attempts to mitigate these by using HCs who resided with their respective PD patient. Analysis of the dietary FFQ indicated that significant macro and micronutrient differences did not exist between the two groups, aside from increased free sugar intake in the PD cohort, which is likely a function of dopamine dysregulation (Palavra et al., 2021). Few prior cross-sectional PD GM studies have evaluated the influences of diet in their analyses but is of potential importance given the beta diversity changes associated with clinical parameters and nutrition identified here. Therefore, we undertook predictive multivariate modeling in an effort to better define the GM as a biomarker for PD. Our results using just the GM (AUC 0.71) were comparable to earlier reports, with AUCs of between 0.64 and 0.84 reported (Scheperjans et al., 2015; Bedarf et al., 2017; Hopfner et al., 2017; Qian et al., 2018). A major point of difference was that in this study we were able to assess the entirety of the GM data at various taxonomic levels, rather than using individual taxa showing statistically significant relative abundance changes. Uniquely, our modeling was expanded and optimized as a two-stage model that incorporated additional clinical and nutritional data, in a manner that most accurately predicts PD. We were able to show that the incorporation of carbohydrate contribution to total energy was an important consideration in the ability to accurately predict PD rather than utilizing GM data alone, resulting in an AUC of 0.74. Future predictive metanalyses of the GM in PD should consider incorporating clinical variables, such as macronutrient data, to optimize PD predictability from GM compositions.

Several limitations to this study should be considered, as it does not address certain potential confounding factors, including other comorbidities and other non-PD medication effects. Medication use for GI dysfunction (e.g., laxatives, anti-diarrhea medication, and reflux medication), as well as GI tract medical diagnosis (e.g., inflammatory bowel disease, inflammatory bowel syndrome and coeliac disease), are important modulators in the GI measures, but were not available and were not considered in the analysis, as reported earlier (Lubomski et al., 2020b). Whether these covariates alter the PD-specific GM profiles is yet to be analyzed. PD and HC groups non-optimally matched for age and sex due to spousal recruitment, may have resulted in confounding in the comparative GM analysis, as age and sex are known to influence GM composition, and are classical matching criteria between case and control groups (Baldini et al., 2020). The utilization of cohabitants or spousal HC is generally more suitable to adjust for geographic and environmental confounders, although differences in age, sex distributions and methodological inconsistencies, may potentially account for some heterogeneity in outcomes and observed GM profiles. This highlights the importance for subsequent studies to have much larger sample sizes, be inclusive of highly diverse cohorts, include comprehensive clinical and nutrition data and have consensus study design to allow for large meta-analyses that may identify more absolute microbial signatures, as have recently been reported (Nishiwaki et al., 2020; Romano et al., 2021). The benefit of larger meta-analysis, particularly greater statistical power, may further allow investigation of smaller subgroups of patients, such as those with younger onset or genetically driven PD, those receiving DATs, as well as thoroughly expose the influences of geography and environmental exposures. Larger multicenter analyses may also reveal why *Lactobacillaceae* and *Bifidobacterium* have been persistently elevated in PD across so many studies, determining if their overexpression is a beneficial compensatory mechanism to overcome dysbiosis or an unfavorable response to increasing disease progression, motor severity and daily LED. The findings presented in this study should be interpreted with consideration for these and other limitations, including the self-reporting nature of the data and potential selection bias of the study population being drawn from specialist PD clinics. Due to the cross-sectional nature of this study, causal inferences were not possible. Furthermore, our sample only reflected the experience of patients from a single metropolitan area, whereas previous studies from Australia have shown PD patients from regional areas to be comparably older with an older age of diagnosis (Lubomski et al., 2013, 2014).

## Conclusion

In our cohort of Australian PD patients and HCs, we showed distinctly differentially abundant bacterial taxa, validating trends from prior studies, as well as identifying new genera that may also be implicated. We identified many new motor and non-motor associations with specific microbiota that create PD-specific GM profiles, in addition to exploring relationships between standard therapies and DATs. We utilized the apparent associations between GM changes in PD with clinical and nutrition features to define a predictive model that could aid clinicians in the diagnosis and management of PD, particularly if developed for prodromal or early disease. Further studies incorporating larger cohorts and targeted subgroups, such as younger onset and pre-clinical patients, are required. In addition, more comprehensive longitudinal studies and meta-analyses are needed to better understand the causal implications of the GM in PD, define therapeutic interventions that favorably modify the GM and develop more accurate predictive models to improve early diagnosis.

## Data Availability Statement

The datasets presented in this study can be found in the NCBI repository under accession number PRJNA808166.

## Ethics Statement

The studies involving human participants were reviewed and approved by the Northern Sydney Local Health District Human Research Ethics Committee (HREC/18/HAWKE/109) and the North Shore Private Hospital ethics committee (NSPHEC 2018-LNR-009). The patients/participants provided their written informed consent to participate in this study.

## Author Contributions

ML conceived and designed the study, recruited, examined all participants, collected, generated and analyzed the data, drafted, and reviewed the manuscript. XX analyzed the genomic and clinical data, drafted, and reviewed the manuscript. AH designed the study, analyzed the data, drafted, and reviewed the manuscript. SM and JY designed the genomic and clinical analysis, drafted, and reviewed the manuscript. RD conceived and designed the study, generated the data, drafted, and reviewed the manuscript. CS conceived and designed the study, drafted, and reviewed the manuscript. All authors contributed to the article and approved the submitted version.

## Conflict of Interest

The authors declare that the research was conducted in the absence of any commercial or financial relationships that could be construed as a potential conflict of interest.

## Publisher’s Note

All claims expressed in this article are solely those of the authors and do not necessarily represent those of their affiliated organizations, or those of the publisher, the editors and the reviewers. Any product that may be evaluated in this article, or claim that may be made by its manufacturer, is not guaranteed or endorsed by the publisher.

## Footnotes

1
http://shiny.maths.usyd.edu.au/PDBug/
