# Supplementary material for: Nutritional Intake and Gut Microbiome Composition Predict Parkinson’s Disease
Source: Front Aging Neurosci. 2022 May 11;14:881872. doi: 10.3389/fnagi.2022.881872 (PMC9131011; doi:10.3389/fnagi.2022.881872)
Supplement: Supplementary file 1 [file Data_Sheet_1.DOCX]

**Nutritional intake and gut microbiome composition predict Parkinson’s disease**

**Supplementary Data**

**Figures:**

**
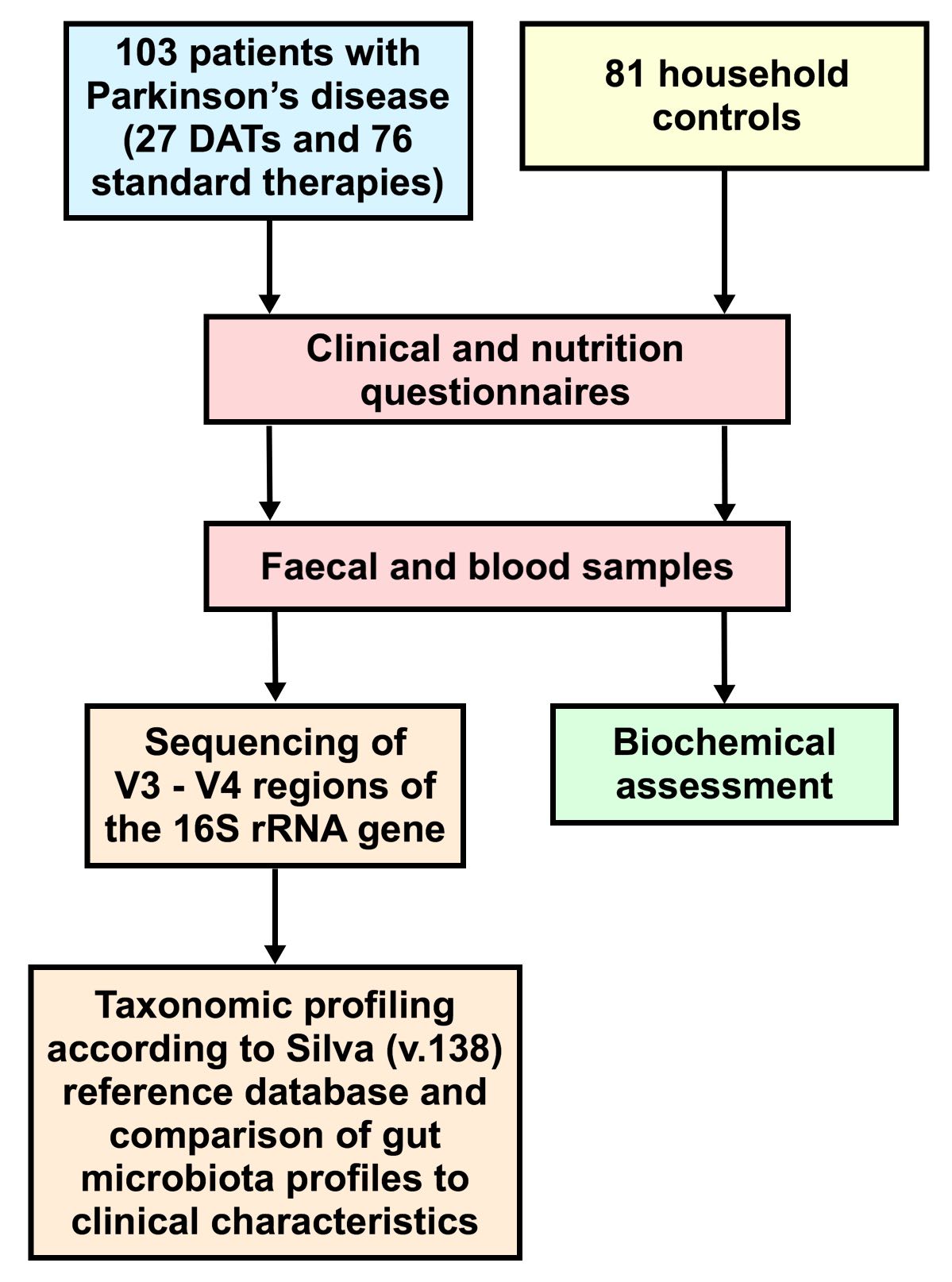
**

**Supplementary Figure 1: Recruitment of Parkinson’s disease (PD) and household control (HC) participants.** Participants completed clinical and nutrition questionnaires and provided faecal biospecimens for 16S amplicon sequencing analysis of the gut microbiome and routine biochemical blood tests. Following 16S sequencing, taxonomic profiling was used to assess differences between the PD (n=103) and HC (n=81) groups, evaluating clinical associations with the gut microbiome in addition to performing predictive modelling.

**
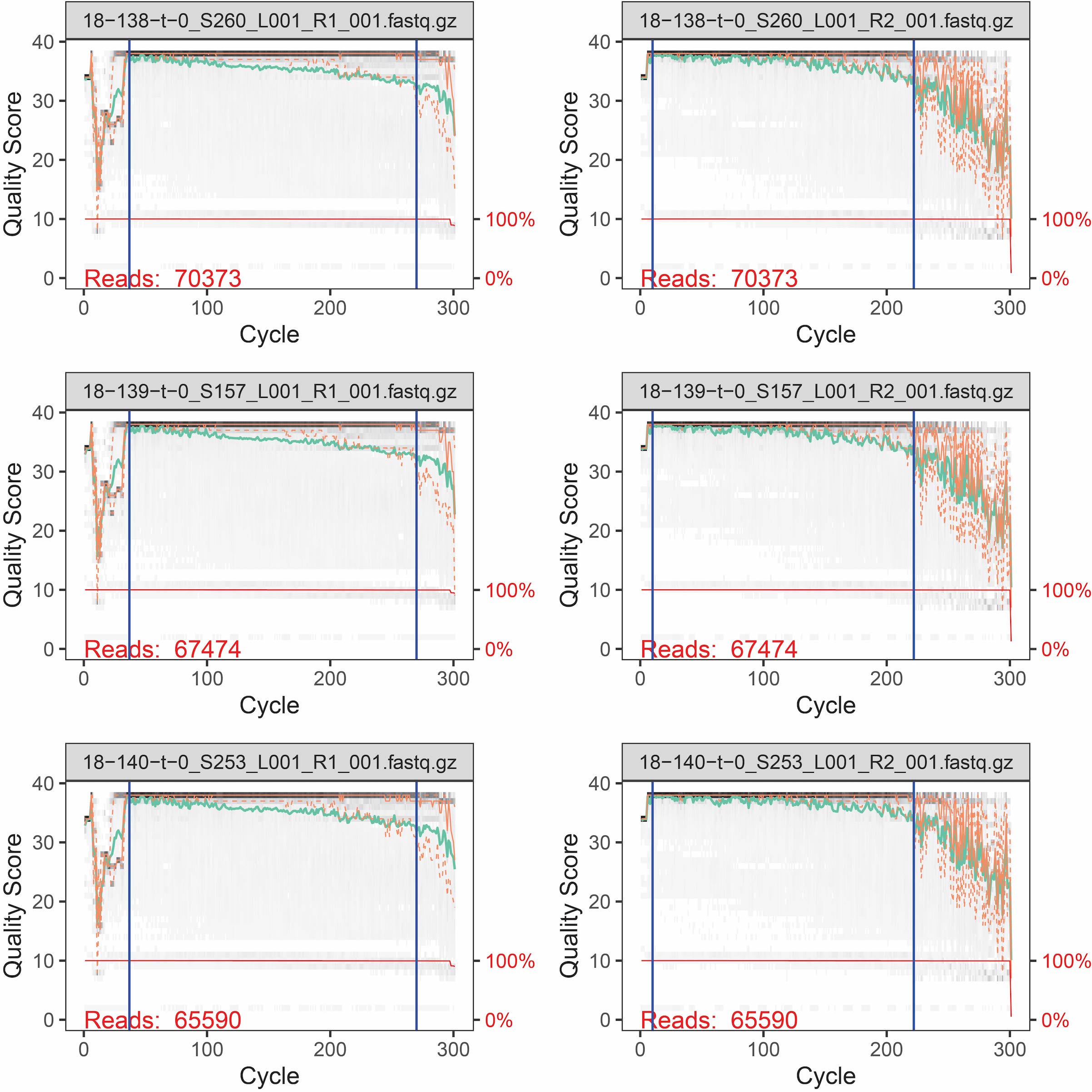
**

**Supplementary Figure 2: 16S amplicon sequencing read trimming and quality control.**

A representative selection of samples showing raw paired sequencing read quality (R1 in the left column, R2 in the right column). Reads were trimmed at either end to between 200 and 300 bases for a final read quality score >30. The total number of reads per sample is shown in red in the bottom left of each plot.


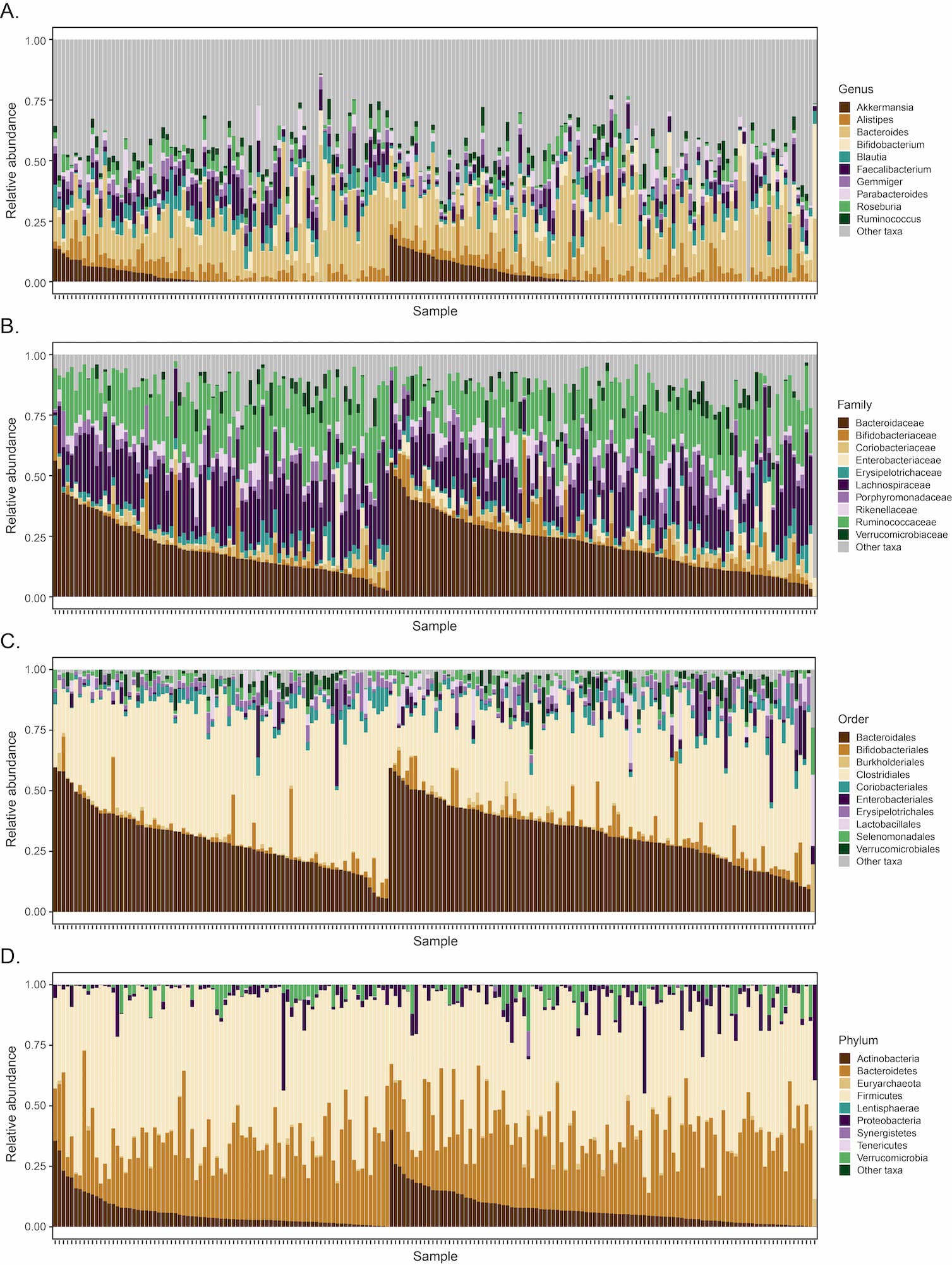


**Supplementary Figure 3: Measures of relative gut bacterial abundance between household control (HC) and Parkinson’s disease (PD) groups at four taxonomic levels.** Relative bacterial abundances are shown for individual HC (left hand side) and PD (right hand side) participants, ordered by the most representative bacteria at A) genus, B), family, C) order and D) phylum levels. At each taxonomic level shown, a mean relative abundance statistical difference was noted between the HC and PD groups, (PERMANOVA, *p*<0.01 genus, *p*<0.01 family, *p*<0.01 order, *p*=0.02 phylum taxonomic levels).


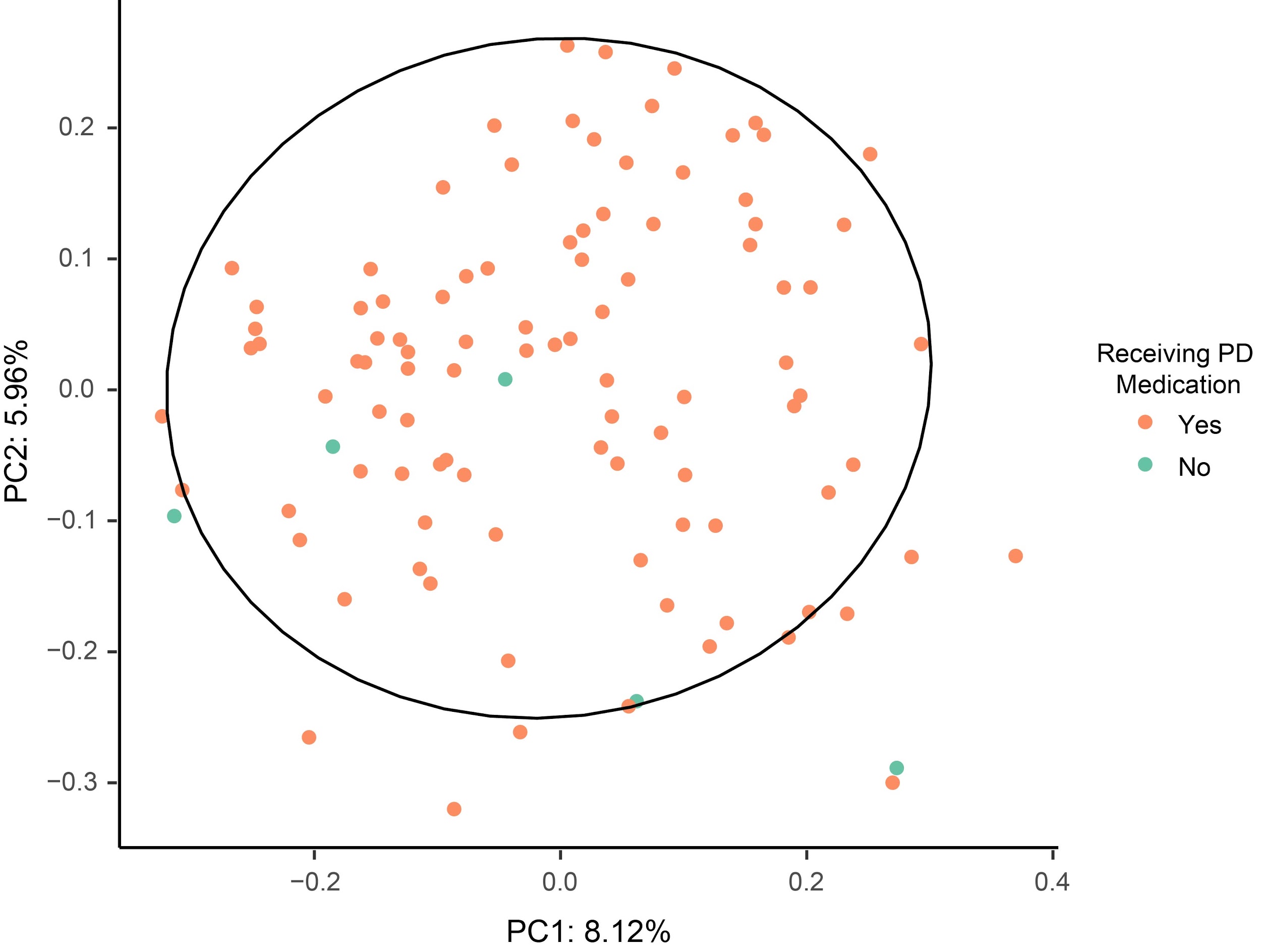


**Supplementary Figure 4: Beta diversity in drug naïve and treated Parkinson’s disease (PD) patients.** PCoA with Bray-Curtis dissimilarity was used to assess beta diversity at the Amplicon Sequencing Variant (ASV) level, comparing PD patients receiving any PD therapy (orange) with patients who were drug naïve (green). Although not statistically different compared to the overall PD cohort, a slight separation was noted in the drug naïve group, suggesting a potential trend in the subgroup analysis, which would require further evaluation in larger cohorts.

**Tables**

**Supplementary Table 1: Statistically significant Associations Between Gut Bacteria and Clinical Variables after adjusting for Age, Sex and Body Mass Index.**

| Clinical variable | Bacteria associating  with clinical variable | Spearman Correlation coefficient (*r_s_*) | *p* value |
| --- | --- | --- | --- |
| Medications and Therapies  *Device-Assisted Therapies*  LCIG (n=9)  DBS (n=11)  Apomorphine Infusions (n=7)  *Standard Therapies*  Levodopa (n=92)  Anticholinergics (n=13)  COMT Inhibitors (n=24)  Amantadine (n=13)  Dopamine Agonists (n=36)  MAO-B Inhibitors (n=19)  Daily Levodopa Dose Equivalence | *Tenericutes*  *Proteobacteria*  *Enterobacteriales*  *Enterobacteriaceae*  *Enterococcus*  *Klebsiella*  *Claoacibacillus*  *Aneroplasma*  ASV_155/*Klebsiella spp*  ASV_512/*Clostridiales spp*  ASV_404/*Blautia spp*  *Streptococcaceae*  *Rikenellaceae*  *Streptococcus*  *Eggerthella*  *Alistipes*  ASV_222/*Streptococcus spp*  ASV_25/ *Streptococcus spp*  ASV_166/*Bifidobacterium spp*  *Intestinibacter*  *Parasutterella*  *Actinomyces*  ASV_611/*Mogibacterium spp*  ASV_529/*Blautia spp*  ASV_382/*Ruminococcus spp*  ASV_381/*Clostridium_sensu_stricto spp*  *Burkholderiales*  *Sutterellaceae*  *Rikenellaceae*  *Alistipes*  *Turicibacter*  *Oscillibacter*  *Dialister*  ASV_166/*Bifidobacterium spp*  ASV_430/*Lactobacillus spp*  *Actinobacteria*  *Bifidobacteriaceae*  *Enterococcaceae*  *Lactobacillaceae*  ASV_139/*Lactobacillus spp*  ASV_512/*Clostridiales spp*  *Anaerofilum*  *Lactobacillus*  ASV_314/*Bacteroides spp*  ASV_605/*Streptococcus spp*  ASV_45/*Intestinibacter spp* ASV_42/*Bifidobacterium spp*  *Enterobacteriales*  *Bifidobacteriales*  *Lactobacillales*  *Lactobacillaceae*  *Enterobacteriaceae*  *Enterococcaceae*  *Bifidobacteriaceae*  ASV_82/*Lactobacillus spp*  ASV_233/*Bifidobacterium spp* | 0.276  0.234  0.284  0.294  0.531  0.366  -0.288  0.274  0.411  0.388  0.369  0.332  -0.280  0.331  0.286  -0.260  0.380  0.375  -0.280  0.307  0.295  0.240  0.375  0.352  0.346  0.341  0.335  0.310  0.310  0.310  0.284  0.283  0.259  0.398  0.354  0.255  0.387  0.284  0.273  0.325  0.314  0.264  0.213  0.284  0.274  -0.264  0.243  0.320  0.264  0.236  0.323  0.300  0.293  0.266  0.398  0.335 | 0.010  0.020  <0.001  <0.001  0.010  <0.001  0.020  0.010  <0.001  <0.001  <0.001  <0.001  0.020  0.001  <0.001  0.020  <0.001  <0.001  0.040  <0.001  0.010  0.040  0.001  <0.001  <0.001  <0.001  <0.001  <0.001  <0.001  <0.001  <0.001  <0.001  0.010  <0.001  <0.001  0.010  <0.001  <0.001  0.010  <0.001  <0.001  0.010  0.030  0.001  <0.001  0.010  0.010  <0.001  0.010  0.020  <0.001  <0.001  <0.001  0.010  <0.001  <0.001 |
| Motor Features and Indicators of Disease Severity  UPDRS-III Score  Hoehn and Yahr Stage  PD Duration  PD Onset > 60 years  PD Onset < 40 years  Postural Instability PD Phenotype  Motor Fluctuations | *Lactobacillales*  *Lactobacillaceae*  *Enterococcaceae*  *Lactobacillus*  *Gordonibacter*  *Enterococcus*  *Eggerthella*  *Escherichia/Shigella*  *Lactobacillaceae*  *Enterobacteriaceae*  *Enterococcaceae*  *Escherichia/Shigella*  *Lactobacillus*  *Eggerthella*  *Enterococcus*  *Lactobacillus*  *Eggerthella*  *Escherichia/Shigella*  *Anaerovorax*  *Klebsiella*  *Bifidobacteriaceae*  *Cloadbacillus*  *Oxalobacter*  *Holdemanella*  *Bifidobacterium*  *Ruminococcus2*  *Dorea*  *Oxalobacter*  *Synergistaceae*  *Oxalobacteraceae*  *Faecaliococcus*  *Escherichia/Shigella*  *Senegalimassillia*  ASV_570/*Ruminococcus spp*  ASV_233/*Bifidobacterium spp*  *Escherichia/Shigella*  *Klebsiella*  *Cloacibacillus* | 0.284  0.257  0.222  0.254  0.234  0.227  0.219  0.216  0.334  0.295  0.272  0.334  0.332  0.323  0.275  0.323  0.244  0.233  -0.233  0.222  -0.216  0.344  0.331  -0.235  -0.218  0.327  0.265  -0.214  0.333  0.304  0.315  0.271  0.212  0.366  0.322  0.336  0.258  -0.244 | <0.001  0.010  0.030  0.010  0.010  0.030  0.030  0.030  <0.001  <0.001  0.010  <0.001  <0.001  <0.001  0.010  <0.001  0.010  0.020  0.020  0.030  0.040  <0.001  <0.001  0.040  0.040  <0.001  0.010  0.030  <0.001  <0.001  <0.001  0.010  0.030  <0.001  <0.001  <0.001  0.010  0.020 |
| Non-Motor Symptoms  Non-Motor Symptoms Scale (Total Score)  Quality of Life  PDQ-39 Summary Index  SF-36 Physical Component Summary  Depression  Beck’s Depression Inventory Score  Physical Activity  IPAQ Score  Chronic Pain  REM Sleep Behaviour Disorder  Gastrointestinal Function  Bristol Stool Scale Score  ROME-IV Score  Leeds Dyspepsia Score | *Pseudoflavonifractor*  *Gordonibacter*  *Gemella*  ASV_570/*Ruminococcus spp*  *Lactobacillaceae*  *Victivallaceae*  *Gordonibacter*  *Eggerthella*  *Pseudoflavonifractor*  *Lactobacillus*  *Oxalobacter*  *Firmicutes*  *Enterobacteriaceae*  *Bacteroidaceae*  *Fusicatenibacter*  *Butyricicoccus*  *Eggerthella*  *Holdemania*  *Pseudoflavonifractor*  ASV_32/*Blautia spp*  ASV_349/*Eggerthella spp*  ASV_476/*Clostridium_XlVa spp*  *Veillonella*  *Klebsiella*  *Pseudoflavonifractor*  *Lactobacillaceae*  *Enterobacteriaceae*  *Lactobacillus*  *Enterobacteriaceae*  *Bacteroidaceae*  *Synergistaceae*  *Veillonella*  ASV_32/*Blautia spp*  *Porphyromonadaceae*  *Gordonibacter*  ASV_151/*Bacteroides spp*  *Butyricicoccus*  *Faecalibacterium*  *Escherichia/Shigella*  *Coprococcus*  ASV_350/ *Clostridium_XlVa spp*  ASV_184/*Clostridium_XlVa spp*  ASV_15/*Faecalibacterium spp*  ASV_46/*Roseburia spp*  *Holdemania*  *Butyricicoccus*  *Romboutsia*  ASV_151/*Bacteroides spp*  ASV_133/*Alistipes spp*  *Desulfomicrobiaceae*  *Desulfomicrobium*  *Mogibacterium* | 0.371  0.243  -0.223  0.244  0.273  -0.235  0.300  0.288  0.281  0.274  -0.211  0.378  -0.240  -0.230  0.444  0.438  -0.340  -0.311  -0.310  0.401  -0.267  -0.256  0.314  0.312  0.311  -0.274  -0.233  -0.275  -0.298  0.288  0.267  -0.368  0.334  0.246  0.302  0.322  0.428  0.364  -0.334  0.318  0.398  0.372  0.371  0.371  0.342  0.321  -0.233  0.411  0.388  0.438  0.422  0.350 | <0.001  0.010  0.040  0.010  0.010  0.020  <0.001  <0.001  <0.001  0.010  0.040  <0.001  0.001  0.002  <0.001  <0.001  <0.001  <0.001  <0.001  <0.001  0.001  0.001  <0.001  <0.001  <0.001  0.001  0.002  0.001  <0.001  <0.001  0.001  <0.001  <0.001  0.020  <0.001  <0.001  <0.001  <0.001  <0.001  <0.001  <0.001  <0.001  <0.001  <0.001  <0.001  <0.001  0.002  <0.001  <0.001  <0.001  <0.001  <0.001 |
| Other Clinical Factors  Increasing Age  Male Gender (n=58)  Ethnicity  Caucasian (n=81)  Asian (n=4)  Prior Neuroleptic Exposure (n=3)  BMI  Dietary Influences  Coffee Consumption  Added Sugars | *Synergistetes*  *Euryarchaeota*  *Lentisphaerae*  *Firmicutes*  *Synergistaceae*  *Methanobacteriaceae*  *Eisenbergiella*  *Cloacibacillus*  *Methanobrevibacter*  ASV_343/*Eisenbergiella spp*  ASV_198/*Cloacibacillus spp*  ASV_453/*Butyricimonas spp*  ASV_609/*Clostridiales spp*  *Euryarchaeota*  *Synergistetes*  *Synergistaceae*  *Methanobacteriaceae*  *Romboutsia*  *Turicibacter*  *Rhodospirillaceae*  *Rikenellaceae*  *Desulfovibrionaceae*  *Synergistaceae*  *Alistipes*  *Desulfovibrio*  ASV_299/*Parabacteroides spp*  ASV_198/*Cloacibacillus spp*  *Gordonibacter*  *Escherichia/Shigella*  *Parasutterella*  ASV_173/*Parasutterella spp*  *Actinomycetaceae*  *Actinomyces*  ASV_545/*Actinomyces spp*  ASV_335/*Parabacteroides spp*  *Anaeroplasma*  *Romboutsia*  *Adlercreutzia*  *Lactobacillus*  *Mogibacterium*  *Streptococcus* | 0.324  0.256  0.222  -0.222  0.321  0.257  0.388  0.364  0.267  0.384  0.363  0.355  0.323  0.294  0.288  0.287  0.295  0.334  0.299  0.367  0.355  0.263  0.248  0.355  0.301  0.366  0.306  0.298  0.277  0.257  0.489  0.383  0.383  0.383  0.373  -0.311  0.288  0.290  0.344  0.293  0.248 | <0.001  0.001  0.030  0.030  <0.001  <0.001  <0.001  <0.001  <0.001  <0.001  <0.001  <0.001  <0.001  <0.001  <0.001  <0.001  <0.001  <0.001  0.001  <0.001  <0.001  <0.001  0.010  <0.001  <0.001  <0.001  <0.001  <0.001  <0.001  0.010  <0.001  <0.001  <0.001  <0.001  <0.001  <0.001  0.010  <0.001  <0.001  <0.001  0.001 |

Comparisons with a moderate Spearman correlation (*r*_s_ = 0.4-0.6) are highlighted in yellow and those with a borderline moderate Spearman correlation (*r*_s_ = 0.398) are highlighted in blue. All other correlations listed are considered weak Spearman correlations (*r*_s_ = 0.2-0.4). Further interactive visualization of the data is available online at: <http://shiny.maths.usyd.edu.au/PDBug/>
